# Modeling of Maternal Factors Affecting Child Ever Born in Punjab, Pakistan: Indication From Multiple Indicator Cluster Survey (2017–2018)

**DOI:** 10.1002/ajhb.70259

**Published:** 2026-04-13

**Authors:** Maryam Siddiqa, Amber Zubair, Asif Hanif, Shazia Iqbal, Tahira Ashraf, Shahzad Ahmad

**Affiliations:** ^1^ Department of Mathematics & Statistics International Islamic University Islamabad Islamabad Pakistan; ^2^ Department of Biostatistics, Faculty of Medicine Sakarya University Sakarya Türkiye; ^3^ Faculty of Medicine and Health Science The University of Buckingham Buckingham UK

**Keywords:** child ever born, children, fertility, Pakistan, stopping behavior

## Abstract

**Objective:**

Fertility trends and population dynamics in Pakistan significantly influence the nation's socioeconomic progress. Elevated fertility rates drive rapid population expansion, creating major challenges for the healthcare system, education sector, and overall resource management. This study aims to identify key determinants influencing household fertility decisions among women aged 15–49 years in Punjab, Pakistan.

**Methods:**

The number of children ever born to a woman is used as a proxy for household fertility. The analysis is based on data from the latest round of the Punjab Multiple Indicator Cluster Survey (MICS) 2017–2018. A Poisson regression model was employed to account for the count nature of the dependent variable. Both Poisson regression and logistic regression analyses were conducted to explore the most significant predictors of fertility variation across the province.

**Results:**

Both the Poisson and logistic regression analyses identified a similar set of significant factors influencing fertility, including age, education, household wealth, fertility intentions, and delivery method. In both models, fertility increased progressively with advancing age, whereas higher educational attainment and better economic status were associated with reduced fertility and lower odds of having more than two children. Women who reported no desire for additional children exhibited higher fertility and a greater likelihood of higher parity in each model, while casarean delivery consistently showed a negative association with fertility outcomes.

**Conclusion:**

Fertility differentials across Punjab appear to be shaped by a combination of biological, socioeconomic, and maternal factors. The study highlights the influence of women's age, household wealth, desire for a child, educational attainment, and previous delivery methods on reproductive behavior. These findings carry important implications for fertility regulation strategies in high‐fertility settings and suggest targeted interventions for promoting planned parenthood in similar socioeconomic contexts.

## 1 Introduction

One of the key indicators used to assess population growth and fertility patterns is the number of children ever born (CEB) to a woman (United Nations Department for Economic and Social Affairs 2023; Bwalya and Odimegwu 2025). This measure serves as a general reflection of a woman's reproductive behavior (Bwalya and Odimegwu 2025). The total number of children a woman bears is influenced by a combination of biological, behavioral, and socioeconomic factors, each of which may exert a positive or negative influence on fertility outcomes (Palamuleni 2023). Both individual‐level attributes and broader community‐level characteristics have been shown to shape reproductive decision‐making among women. The fertility‐inhibiting behavior framework is comprised of initiation, spacing, and cessation of childbearing, particularly, relevant during the early stages of fertility transition (Bwalya and Odimegwu 2025; Palamuleni 2023; Tessema and Tamirat 2020; Pal and Shekhar 2021). “Starting behavior” refers to the timing of first reproduction, which is often linked to age at marriage, especially in societies with strong marital norms. “Spacing behavior” refers to the intervals between successive births, with longer intervals generally contributing to reduced fertility. “Stopping behavior” describes the decision to cease childbearing after achieving the desired family size. Of these, stopping behavior played a more prominent role than spacing during early fertility transitions in Europe, largely due to the availability of clearer measurement indices (Knodel 1987; Bavel 2004).

Fertility rates are crucial demographic indicators for understanding population dynamics (Bwalya and Odimegwu 2025; Muchtar and Purnomo 2009). CEB, defined as the total number of live births to ever‐married women aged 15–49, provides an important metric for analyzing population growth, as it is a cumulative measure (Kiser and Hossain 2019; Bongaarts and Casterline 2013; Saadati 2015). Beyond its use as a fertility indicator, CEB is integral to understanding shifts in population structure and age composition (Bbaale and Mpuga 2011; Barkat‐e‐Khuda and Hossain 1996; Fazle Rabbi and Kabir 2015). It is widely acknowledged that biological, behavioral, and socioeconomic factors significantly impact reproductive behavior (Younglai et al. 2005), and community‐level variables can also influence women's fertility decisions (Bwalya and Odimegwu 2025; Yebyo et al. 2015; Gebre 2024). Both take from folder.

The global population has expanded to more than three times its size in the 1950s and is anticipated to reach a peak of nearly 11 billion by the year 2100 (United Nations 2022; United Nations Department of Economic and Social Affairs PD 2022). A substantial portion of this growth is projected to occur within low‐income and lower‐middle‐income nations (United Nations 2022; United Nations Department of Economic and Social Affairs PD 2022). Specifically, eight countries: India, Nigeria, Pakistan, the Democratic Republic of the Congo, Ethiopia, Egypt, the Philippines, and the United Republic of Tanzania are expected to contribute to over half of the total projected population increase by 2050 (United Nations Department of Economic and Social Affairs PD 2022). This significant demographic expansion since 1950 has primarily resulted from a gradual rise in average life expectancy and the sustained high fertility rates in several regions (United Nations 2022). As long as fertility remains elevated, global population growth is expected to continue (United Nations 2022; United Nations Department of Economic and Social Affairs PD 2022).

In Pakistan, rapid population growth presents a serious obstacle to economic development. Pakistan was one of the first countries to establish fertility regulation and control programs as far back as the 1950s. However, such programs were limited in their effectiveness due to cultural constraints and religious ideologies (Wazir et al. 2021). By 2020, Pakistan had emerged as the fifth most populous country globally (Lai 2021). From a population growth rate of approximately 1% between 1951 and 1980, growth jumped to 3% as of 2017 (Qasim et al. 2023). The country's TFR declined from 4.9 before the 1990s to 4.1 by 2006 (Javed and Mughal 2022), while remaining higher compared to neighboring countries such as Bangladesh and India (Lai 2021).

Although various policy efforts have been introduced over the years, the total fertility rate (TFR) has remained a significant concern. Globally, fertility rates have shown a downward trend over recent decades. Between 1990 and 2019, the global TFR declined from 3.2 to 2.5. However, this trend has not been uniform across regions of the world (United Nations 2020). According to the Pakistan Demographic and Health Survey (PDHS) 2017–2018, the average number of children per woman is 3.6 nationally, while Punjab reports a slightly lower TFR of 3.4 (National Institute of population studies (NIPS) Pakistan and ICF International 2019). Since 1990–1991, the national TFR has declined from 4.9 to 3.6, a reduction of 1.3 children per woman (National Institute of population studies (NIPS) Pakistan and ICF International 2019). However, the most recent Maternal mortality DHS (2019) suggests a slight increase, placing the TFR at 3.7, indicating a potential reversal or stagnation in the fertility decline trend in Pakistan (Pakistan Bureau of Statistics 2020).

Education and urbanization are among the most frequently cited determinants of childbearing behavior (Bwalya and Odimegwu 2025; Afreen et al. 2024) and an inverse relationship between maternal education and the average number of CEB was reported. Existing literature consistently identifies factors such as maternal age, place of residence, administrative region, and exposure to mass media as being significantly associated with fertility levels among ever‐married women (Saadati 2015; Gebre 2024). Other important determinants include age at first birth, contraceptive use, educational attainment of both the woman and her spouse, religious affiliation, and household wealth index (Bwalya and Odimegwu 2025; Gebre 2024; Karimuzzaman et al. 2020; Götmark and Andersson 2020; Saleem et al. 2025). Numerous studies have found that higher age at marriage and greater wealth are associated with fewer children born (Gebre 2024). Among the various proximate determinants, contraceptive use stands out as a particularly effective method for reducing fertility rates (Gebre 2024; Karimuzzaman et al. 2020). In Bangladesh, employment status and food security have also been shown to influence childbearing decisions (Saleem et al. 2025). A range of factors including age at first marriage and birth, place of residence, media exposure, religion, spousal fertility preferences, women's empowerment, and socioeconomic status have been statistically linked to fertility levels (Bwalya and Odimegwu 2025; Gebre 2024; Saleem et al. 2025; Rahman et al. 2022). Moreover, higher educational attainment among both women and their spouses is typically associated with a lower number of children (Rahman et al. 2022). Elevated CEB has been observed among women who marry before age 18, are illiterate, live in rural areas, have their first child before age 20, lack media exposure, and whose husbands desire more children (Rahman et al. 2022). In Oman, age, education, household structure, and contraceptive use were identified as the primary determinants of fertility levels (Al‐Balushi et al. 2020). Interestingly, no significant differences in fertility were observed between rural and urban settings or across different economic strata in Oman (Al‐Balushi et al. 2020). This comparative analysis investigates the number of CEB to women in Pakistan, with a particular focus on how sociodemographic and maternal characteristics influence reproductive behavior.

## 2 Material and Methods

### 2.1 Study Population and Sample Size

The data for this study were obtained from the Multiple Indicator Cluster Survey (MICS) conducted in Punjab during 2017–2018. Technical support for the survey was provided by the United Nations Children's Fund (UNICEF). Notably, this was the first time that data collection in Punjab was carried out using Computer‐Assisted Personal Interviewing (CAPI). The MICS 2017–2018 survey employed a two‐stage stratified sampling design. Within each district, the primary strata were divided into urban and rural areas. In the first stage, a predetermined number of census enumeration areas (EAs) were selected systematically using probability proportional to size. In the second stage, 20 households were systematically drawn from each selected EA, based on the Pakistan Bureau of Statistics' household listings from the 2017 Census. The survey targeted total of 53 480 household selected at the second stage and questionnaire information for all individuals women of each household aged 15–49 was taken and around 74 010 women were interviewed.

This study was conducted in accordance with the ethical principles outlined in the Declaration of Helsinki. Ethical clearance was obtained from the Institutional Review Committee of the Islamic International University, Islamabad, Pakistan (IIU ORIC Bioethics Committee; Protocol No. 110, dated April 22, 2022). As this study utilized publicly available, deidentified secondary data, informed consent from participants was not applicable.

### 2.2 Response Variable

The outcome variable “number of children ever born” (CEB) to a woman during her life was used as a count variable in Poisson regression analysis. The outcome variable “number of children ever born” (CEB) was recoded into a binary variable for logistic regression analysis, where a value of 0 indicates two or fewer children (≤ 2), and a value of 1 represents more than two children (> 2).

### 2.3 Independent Variables

The independent variables included in the analysis encompassed a range of sociodemographic and maternal characteristics. These were age of the respondent (categorized as 15–19, 20–24, 25–29, 30–34, 35–39, 40–44, and 45–49 years), level of education (none, primary, secondary, and higher), and household wealth status (poor, middle, rich). Maternal factors included history of previous delivery by casarean section (yes, no), ever breastfed (yes, no), desire of child (right now, no more), mother age at marriage (10–14, 15–19,20–24, 25–29, 30–34, 35–39, 40–44, 45–49). Additional variables were: ever used the internet (yes, no), gender of the preceding child (boy, girl), area of residence (urban, rural), number of preceding births (≤ 2, > 2), age of husband (15–19, 20–24, 25–29, 30–34, 35–44, 45–49, ≥ 50), ever used contraceptive method (no, yes), currently used contraceptive method (no, yes), current contraceptive method (traditional, modern), current fertility intention (no, yes), and spacing intention (no, yes).

Other variables regarding contraceptive use in the study are “ever used contraceptive method” categorized as (no vs. yes), “currently used contraceptive method” (no vs. yes), and “current contraceptive methods” was categorized as (traditional vs. modern). The variable representing current contraceptive methods was operationalized dichotomously. Respondents who reported the use of modern contraceptive methods including male or female sterilization, intrauterine devices (IUDs), implants, oral contraceptive pills, male or female condoms, and injectable contraceptives were coded as “1.” In contrast, those who relied on traditional contraceptive practices, such as the lactational amenorrhea method, withdrawal, periodic abstinence, as well as individuals not using any form of contraception, were coded as “0.” The birth “spacing intention” variable was categorized as “Yes, wants to space (after 2+ years)” and “No, wants soon (within 2 years),” while the “fertility intention/desire for child” variable was dichotomous measure as “yes = right now” were coded “1” and “no = no more” were coded “0” was designed to distinguish women who currently desire an additional child from those who have completed their desired family size. This approach facilitates comparability with previous studies and ensures adequate statistical power within categories, thereby enhancing the robustness and interpretability of the analysis. These coding decisions were informed by both theoretical relevance and methodological considerations. The dichotomization of birth spacing into “yes” and “no” was intended to capture the presence or absence of any practice of spacing between births, a categorization approach widely adopted in demographic and reproductive health research to reflect behavioral outcomes in a clear and interpretable manner. This categorization approach ensures clarity in interpretation, maintains adequate sample sizes within categories, facilitates comparability with previous studies and aligns with standard practices in fertility and population studies of MICS and related surveys.

### 2.4 Statistical Analysis

After wrangling of data, survey weights were incorporated appropriately in our analysis of the MICS data. Specifically, the svyset command in STATA was used to account for the complex survey design by generating scaled weights by using the individual weight variable V005 provided in the women file. Descriptive statistics for the number of CEB were summarized using frequency distributions across selected explanatory variables as a count and binary variable simultaneously. To examine associations between the outcome variable and independent variables, both unadjusted bivariate analyses and an adjusted Poisson regression model and binary logistic regression models were employed.

The number of CEB to women aged 15–49 years served as the dependent variable to assess fertility behavior. Given the count nature of the dependent variable CEB, a Poisson regression model was initially applied as the baseline model. The assumption of equi‐dispersion was tested. Initially Poisson regression model is run to check over dispersion. The over‐dispersion test, Pearson–*χ* ^2^, and Hosmer–Lemeshow test displayed the presence of under‐dispersion in the data, justifying the continued use of the Poisson model for analysis. Therefore, the standard Poisson regression model was deemed appropriate for the final analysis of the count response variable.

Due to the count nature of the outcome variable and the presence of under‐dispersion, the Poisson regression model was considered an appropriate choice for modeling fertility outcomes as the primary analytical technique. To complement this, a secondary analysis was conducted using logistic regression, in which fertility was dichotomized into a threshold of ≤ 2 births (low fertility) versus > 2 births (high fertility). Consistent with national population policy promoting a two‐child norm, this study formulated hypotheses to assess the association between key sociodemographic factors and fertility outcomes. For analytical clarity, the variable “total children ever born” was dichotomized into two categories: women with fewer than two children and those with more than two. The reason for this categorization was national population policy of Pakistan which promotes two children per woman. This dual modeling strategy provides complementary perspectives: the Poisson model estimates the determinants of the number of CEB, while the logistic model identifies factors associated with exceeding a fertility threshold, thereby addressing policy‐relevant questions such as the drivers of high fertility among women. SPSS (Version 20; IBM Corp., Armonk, NY) and STATA (Version 13; StataCorp LLC, College Station, TX) were used for the analysis.

## 3 Results

### 3.1 Descriptive Statistics

Percentage distribution of study participants across various categories of socioeconomic and maternal characteristics is reported in Table 1.

**TABLE 1:** Descriptive statistics for sociodemographic and maternal characteristics of the women aged (15–49) for Child Eve Born in Punjab Province, Pakistan, MICS (2017–2018).

Variables	Child ever born (count variable)		Child ever born		
	Categories	Frequency (%)	≤ 2	> 2	Total
Age of women	15–19	14 504 (19.60)	14 471 (19.6)	33 (0.04)	14 504 (19.60)
	20–24	13 568 (18.33)	12 746 (17.22)	822 (1.11)	14 504 (19.60)
	25–29	12 642 (17.08)	8806 (11.90)	3836 (5.18)	13 568 (18.33)
	30–34	10 588 (14.31)	4372 (5.91)	6216 (8.40)	12 642 (17.08)
	35–39	9737 (13.16)	2387 (3.22)	7350 (9.93)	10 588 (14.31)
	40–44	7153 (9.66)	1381 (1.86)	5772 (7.80)	9737 (13.16)
	45–49	5818 (7.86)	931 (1.26)	4887 (6.60)	7153 (9.66)
Education of woman	None	1077 (2.20)	545 (0.74)	532 (0.72)	1077 (2.20)
	Primary	14 154 (28.87)	8375 (11.31)	5779 (7.81)	14 154 (28.87)
	Middle	20 221 (41.24)	1460 (1973)	5614 (7.58)	20 221 (41.24)
	Higher	13 579 (27.69)	11.278 (15.23)	2301 (3.11)	13 579 (27.69)
Socioeconomic status	Poor	29 006 (39.19)	15 967 (21.57)	13 039 (17.61)	29 006 (39.19)
	Middle	16 267 (21.98)	10 202 (13.78)	6065 (8.19)	16 267 (21.98)
	Rich	28 737 (38.83)	18 925 (25.57)	9812 (13.26)	28 737 (38.83)
Previous delivery by C‐section	No	7052 (61.39)	3207 (27.93)	3845 (33.46)	7052 (61.39)
	Yes	4435 (38.6)	2618 (22.79)	1817 (15.82)	4435 (38.6)
Ever breastfed	No	1253 (8.0)	595 (3.79)	658 (4.19)	1253 (8.0)
	Yes	14 460 (92.0)	6619 (42.11)	7841 (49.91)	14 460 (92.0)
Desire of child	Later	1343 (46.8)	709 (24.70)	634 (22.09)	1343 (46.8)
	No more	1528 (53.2)	133 (4.63)	1395 (48.60)	1528 (53.2)
Considering importance of spacing	No	10 992 (78.6)	5339 (38.19)	5653 (40.45)	10 992 (78.6)
	Yes	2984 (21.4)	1361 (9.73)	1623 (11.61)	2984 (21.4)
Gender of preceding child	Boy	23 124 (53.78)	7544 (17.53)	15 580 (36.21)	23 124 (53.78)
	Girl	19 908 (6.26)	6572 (15.27)	13 336 (30.98)	19 908 (6.26)
Age at first marriage	10–14	395 (11.63)	75 (2.21)	320 (9.43)	395 (11.63)
	15–19	1808 (15.34)	397 (11.69)	1411 (41.55)	1808 (15.34)
	20–24	850 (25.03)	255 (7.51)	595 (17.53)	850 (25.03)
	25–29	249 (7.33)	97 (2.86)	152 (4.48)	249 (7.33)
	30–34	79 (2.33)	37 (1.09)	42 (1.24)	79 (2.33)
	35–39	13 (0.38)	7 (0.21)	6 (0.18)	13 (0.38)
	40–44	2 (0.06)	1 (0.03)	1 (0.03)	2 (0.06)
Number of preceding births	≤ 2	37 784 (52.69)	23 202 (32.37)	14 582 (20.34)	37 784 (52.69)
	> 2	33 920 (47.31)	20 691 (28.86)	13 229 (18.44)	33 920 (47.31)
Area of residence	Urban	23 630 (29.78)	14 846 (18.70)	8784 (11.07)	23 630 (29.78)
	Rural	55 730 (70.22)	33 562 (42.29)	22 168 (27.94)	55 730 (70.22)
Age of husband	10–19	432 (0.92)	419 (0.89)	13 (0.03)	432 (0.92)
	20–24	2664 (5.66)	2422 (5.15)	242 (0.51)	2664 (5.66)
	25–29	6827 (14.51)	5099 (10.84)	1728 (3.67)	6827 (14.51)
	30–34	8596 (18.27)	4680 (9.95)	3916 (8.32)	8596 (18.27)
	35–39	11 397 (24.22)	3619 (7.69)	7778 (16.52)	11 397 (24.22)
	40–44	5007 (10.64)	971 (2.06)	4036 (8.58)	5007 (10.64)
	45–49	6061 (12.88)	1004 (2.13)	5057 (10.74)	6061 (12.88)
	≥ 50	6072 (12.90)	940 (2.00)	5132 (10.91)	6072 (12.90)
Ever used Internet	No	64 858 (90.76)	37 700 (52.80)	27 158 (38.00)	64 858 (90.76)
	Yes	6603 (9.24)	5086 (7.11)	1517 (2.12)	6603 (9.24)
Ever used contraceptive method	No	27 933 (89.30)	14 922 (47.69)	13 011 (41.60)	27 933 (89.29)
	Yes	3348 (10.70)	925 (2.96)	2423 (7.74)	3348 (10.71)
Currently used contraceptive method	No	26 459 (62.75)	12 627 (29.94)	13 832 (32.81)	26 459 (62.75)
	Yes	15 722 (37.25)	3289 (7.80)	12 433 (29.45)	15 722 (37.25)
Current contraceptive method	Traditional	921 (6.63)	370 (2.66)	551 (3.96)	921 (6.63)
	Modern	12 980 (93.37)	2458 (17.69)	10 522 (75.69)	12 980 (93.37)
Current fertility intention	No	3988 (82.70)	359 (7.45)	475 (9.85)	3988 (82.70)
	Yes	834 (17.30)	2863 (59.38)	1125 (23.34)	834 (17.30)
Spacing intention	No	4408 (28.33)	3726 (23.94)	682 (4.38)	4408 (28.33)
	Yes	11 154 (71.67)	8162 (52.45)	2992 (19.22)	11 154 (71.67)

Descriptive statistics in Table 1 presented that around 19% women had age between 15–19 years. A high proportion of the mothers (38%) were categorized as rich. Around 61% of women never had a previous delivery by C‐section. Majority of mothers (92%) breastfed their infants. About 70% mothers were residing in rural area. Fifty‐three percent of mothers have a boy as their previous child's gender. About 37.3% mother are using a contraceptive method. Overall, 17.3% of women said the pregnancy was not wanted at the time. Overall, modern contraceptive methods are much more common 93.4% than traditional methods 6.6%.

### 3.2 Regression Results

Table 2 shows the poison and logistic univarate regression analysis of child ever born for each of the explanatory variables. The independent variables which are statistically significant with child ever born are age of women, area of resident, ever used Internet, wealth status, education of women previous delivery by C‐section. All other explanatory variables number of preceding births, ever breastfed, considering importance of spacing, gender of preceding child, and fertility intention were proved insignificantly associated with child ever born in unadjusted regression analysis.

**TABLE 2:** Univariate analysis of the sociodemographic and maternal characteristics of the women aged (15–49) for Child Ever Born in Punjab Province, Pakistan, MICS (2017–2018).

Variables	Attribute	IRR (95% CI)	*p*	OR (95% CI)	*p*
Age of women	15–19^a^	—	—	—	—
	20–24	10.017 (9.312–10.775)	< 0.001	28.27 (19.952–40.081)	< 0.001
	25–29	30.024 (27.973–32.225)	< 0.001	191.01 (135.46–269.35)	< 0.001
	30–34	52.027 (48.518–55.855)	< 0.001	632.45 (442.0–879.22)	< 0.001
	35–39	69.228 (64.530–74.269)	< 0.001	1350.02 (956.5–1905.9)	< 0.001
	40–44	78.909 (73.533–84.678)	< 0.001	1832.7 (1295.9–2591.9)	< 0.001
	45–49	87.263 (81.298–93.664)	< 0.001	2301.8 (1624.1–3262.1)	< 0.001
Area of residence	Urban^a^	—	—	—	—
	Rural	1.132 (1.108–1.155)	< 0.001	1.116 (1.081–1.151)	< 0.001
Desire of child	Right now^a^	—	—	—	—
	No more	1.792 (1.723–1.864)	< 0.001	11.729 (9.530–14.436)	< 0.001
Ever used Internet	No^a^	—	—	—	—
	Yes	0.543 (0.524–0.563)	< 0.001	1.071 (0.954–1.202)	0.244
Socioeconomic status	Poor^a^	—	—	—	—
	Middle	0.787 (0.777–0.797)	< 0.001	0.727 (0.699–0.757)	< 0.001
	Rich	0.694 (0.687–0.702)	< 0.001	0.634 (0.613–0.656)	< 0.001
Education of woman	None^a^	—	—	—	—
	Primary	0.807 (0.766–0.838)	< 0.001	0.706 (0.624–0.800)	< 0.001
	Middle	0.542 (0.522–0.563)	< 0.001	0.393 (0.348–0.445)	< 0.001
	Higher	0.344 (0.330–0.358)	< 0.001	0.209 (0.183 0.237)	< 0.001
Number of preceding births	≤ 2^a^	—	—	—	—
	> 2	0.993 (0.982–1.002)	0.159	1.017 (0.987–1.048)	0.263
Ever breastfed	No^a^	—	—	—	—
	Yes	1.000 (0.965–1.037)	0.971	1.071 (0.954–1.202)	0.244
Gender of preceding child	Boy^a^	—	—	—	—
	Girl	1.005 (0.994–1.016)	0.267	0.982 (0.943–1.023)	0.393
Considering importance of spacing	No^a^	—	—	—	—
	Yes	1.021 (0.995–1.046)	0.079	1.126 (1.038–1.221)	0.004
Previous delivery by C‐section	No^a^	—	—	—	—
	Yes	0.803 (0.784–0.822)	< 0.001	0.578 (0.536–0.624)	< 0.001
Age of husband	10–19^a^	—	—	—	—
	20–24	2.004 (1.745–2.302)	< 0.001	3.220 (1.825–5.680)	< 0.001
	25–29	3.293 (2.879–3.767)	< 0.001	10.92 (6.272–19.020)	< 0.001
	30–34	4.847 (4.240–5.541)	< 0.001	26.96 (15.50–46.91)	< 0.001
	35–39	6.882 (6.022–7.864)	< 0.001	69.27 (39.83–120.47)	< 0.001
	40–44	8.217 (7.188–9.393)	< 0.001	133.9 (76.80–233.69)	< 0.001
	45–49	9.066 (7.933–10.362)	< 0.001	162.3 (93.09–283.1)	< 0.001
	≥ 50	9.499 (8.311–10.856)	< 0.001	175.09 (100.8–306.93)	< 0.001
Age at first marriage	10–14	—	—	—	—
	15–19^a^	0.902 (0.860–0.947)	0.903	0.833 (0.632–1.096)	0.193
	20–24	0.759 (0.718–0.802)	0.183	0.546 (0.408–0.731)	< 0.001
	25–29	0.652 (0.601–0.707)	< 0.001	0.367 (0.256–0.525)	< 0.001
	30–34	0.648 (0.569–0.737)	< 0.001	0.266 (0.160–0.442)	< 0.001
	35–39	0.526 (0.376–0.734)	0.024	0.200 (0.065–0.615)	< 0.001
	40–44	0.293 (0.094–0.909)	0.103	0.234 (0.014–3.789)	< 0.001
Currently used contraceptive method	No	—	—	—	—
	Yes	1.388 (1.373–1.402)	< 0.001	3.450 (3.297–3.611)	< 0.001
Ever used contraceptive method	No	—	—	—	—
	Yes	1.432 (1.406–1.459)	< 0.001	0.440 (0.397–0.484)	< 0.001
Current contraceptive method	No	—	—	—	—
	Yes	1.243 (1.199–1.290)	< 0.001	2.874 (2.501–3.302)	< 0.001
Current fertility intention	No	—	—	—	—
	Yes	1.786 (1.708–1.868)	< 0.001	3.367 (2.888–3.925)	< 0.001
Spacing intention	No	—	—	—	—
	Yes	1.678 (1.626–1.732)	< 0.001	2.002 (1.827–2.195)	< 0.001

Abbreviations: CI = confidence interval; IRR = incidence rate ratio; OR = odds ratio.

^a^ Reference category.

Poisson univarate analysis shows women being in the age group of 20–24 or higher age groups (IRR, 10.017; 95% CI, 9.312–10.775), (IRR, 30.024; 95% CI, 27.973–32.225), (IRR, 52.027; 95% CI, 48.518–55.855), (IRR, 52.228; 95% CI, 64.530–74.269), (IRR,78.909; 95% CI, 73.533–84.678), (IRR, 87.263; 95% CI, 81.298–93.664) resulted in a significantly higher expected continuity of childbearing than for women under age 20. Women residing in rural areas (IRR, 1.132; 95% CI, 1.108–1.155) had significantly higher expected child even born than urban areas residents. Secondary and highly‐ educated women (IRR, 0.807; 95% CI, 0.766–0.838) (IRR, 0.542; 95% CI, 0.522–0.563) (IRR, 0.344; 95% CI, 0.330–0.358) had a significantly lower expected childbearing than the women who had no education. The women belonged to high wealth status family (IRR, 0.787; 95% CI, 0.767–0.807) (IRR, 0.694; 95% CI, 0.679–0.710) had lower expected number of children as compared to poor families. Women who used the Internet (IRR, 0.543; 95% CI, 0.524–0.563) had significantly lower number of child ever born than those women who did not use the Internet. Women who had their previous babies by C‐section (IRR, 0.803; 95% CI, 0.784–0.822) had significantly lower expected number of CEB than those who had normal delivery. Women who said no desire of more children had (IRR, 1.792; 95% CI, 1.723–1.864) significantly higher number of child ever born as compared to who said later. Husbands aged 20–24 years or older (IRR, 1.396; 95% CI, 1.285–1.515), (IRR, 1.827;95% CI, 1.687–1.977), (IRR, 2.291;95% CI, 2.117–2.479), (IRR, 2.898; 95% CI, 2.679–3.134), (IRR, 3.478 95%; CI, 3.214–3.763), (IRR, 3.924; 95% CI, 3.627–4.244), (IRR, 4.248; 95% CI, 3.927–4.595) were significantly more likely to be associated with a higher expected rate of childbearing compared with those under age 20. Women who married at ages 25–29 years (IRR, 1.12; 95% CI, 1.04–1.20), 30–34 years (IRR, 1.25; 95% CI, 1.13–1.38), and 35–39 years (IRR, 1.29; 95% CI, 1.03–1.60) had significantly higher expected wage rates than those married before age 15.

Bivariate logistic analysis shows being in the age group of 20–24 or higher age groups (OR, 28.87; 95% CI, 19.952–40.081) (OR, 191.01; 95% CI, 135.46–269.35), (OR, 632.4; 95% CI, 442.0–879.2), (OR, 1350.02; 95% CI, 956.5–1905.9), (OR, 1832.7;95% CI, 1295.9–2591.9), (IRR, 2301.8; 95% CI, 1624.1–3262.1) resulted in a significantly higher expected continuity of childbearing than for women under age 20. Women residing in rural areas (OR, 1.116; 95% CI, 1.108–1.151) had significantly higher expected child even born than urban areas residents. Secondary and highly educated women (OR, 0.706; 95% CI, 0.624–0.800) (OR, 0.393; 95% CI, 0.348–0.445) (OR, 0.209; 95% CI, 0.183–0.237) had a significantly lower expected childbearing than the women who had no education. The women belonged to high wealth status family (OR, 0.727; 95% CI, 0.699–0.757) (OR, 0.634; 95% CI, 0.613–0.656) had lower expected number of children as compared to poor families. Women who had their previous babies by C‐section (OR, 0.578; 95% CI, 0.536–0.624) had significantly lower expected number of CEB than those who had normal delivery. Women who said no desire for more children had (OR, 11.792; 95% CI, 9.530–14.436) significantly higher number of children ever born as compared to who said later. Women who reported that birth spacing is important had higher odds of having more than two children compared with those who did not consider spacing important (OR, 1.13, 95% CI, 1.04–1.22). Women who married at ages 20–24 years (OR, 0.55; 95% CI, 0.41–0.73), 25–29 years (OR, 0.37; 95% CI, 0.26–0.53), 30–34 years (OR, 0.27; 95% CI, 0.16–0.44), and 35–39 years (OR, 0.20; 95% CI, 0.07–0.62) were progressively less likely to have higher parity than those who married before age 15. Women who were currently using a contraceptive method had 3.45 times higher odds of having more than two children compared with non‐users (OR = 3.45; 95% CI = 3.30–3.61). Women who had ever used a contraceptive method had three times higher odds of having more than two children compared with those who had never used any contraceptive method (OR = 3.00; 95% CI = 2.78–3.25). Women who were using modern contraceptive methods had 2.87 times higher odds of having more than two children compared with those using traditional methods (OR = 2.87; 95% CI = 2.50–3.30). Women who intend to have more children have 3.37 times higher odds of having more than two children compared with women who do not intend to have more children (OR = 3.37; 95% CI = 2.89–3.93). Women who intend to space their next birth have 2.00 times higher odds of having more than two children compared with those who do not intend to space or delay childbirth (OR = 2.00; 95% CI = 1.83–2.20).

A multivariable Poisson regression model and binary Logistic Regression model of the CEB of the women of Punjab, Pakistan is presented in Table 3.

**TABLE 3:** Poisson and logistic regression model of the children ever born of the women aged 15–49 Punjab Province, Pakistan, MICS (2017–2018).

Variables	Attribute	AIRR (95% CI)	*p*	AOR (95% CI)	*p*
Age of women	15–19^a^	—	—	—	—
	20–24	1.369 (1.019–1.838)	0.037	4.344 (0.892–21.138)	0.049
	25–29	1.809 (1.359–2.409)	< 0.001	11.92 (2.494–57.042)	< 0.001
	30–34	2.253 (1.690–3.003)	< 0.001	20.17 (4.165–97.764)	< 0.001
	35–39	2.565 (1.918–3.430)	< 0.001	43.92 (8.491–227.19)	< 0.001
	40–44	2.789 (2.050–3.793)	< 0.001	18.13 (2.992–109.85)	< 0.001
	45–49	3.340 (2.196–5.080)	< 0.001	21.33 (3.534–110.23)	< 0.001
Area of residence	Urban^a^	—	—	—	—
	Rural	0.961 (0.896–1.030)	0.265	0.849 (0.615–1.172)	0.321
Desire of child	Right now^a^	—	—	—	—
	No More	1.406 (1.316–1.502)	< 0.001	5.255 (3.862–7.149)	< 0.001
Ever used Internet	No^a^	—	—	—	—
	Yes	1.012 (0.914–1.121)	0.809	1.160 (0.750–1.792)	0.504
Socioeconomic status	Poor^a^	—	—	—	—
	Middle	0.872 (0.799–0.953)	0.002	0.681 (0.423–1.095)	0.113
	Rich	0.845 (0.778–0.917)	< 0.001	0.495 (0.315–0.778)	0.002
Education of woman	None^a^	—	—	—	—
	Primary	0.792 (0.662–0.946)	0.010	0.370 (0.100–1.364)	0.136
	Middle	0.757 (0.632–0.908)	0.003	0.297 (0.080–1.101)	0.070
	Higher	0.667 (0.551–0.807)	< 0.001	0.183 (0.048–0.698)	0.013
Number of preceding births	≤ 2^a^	—	—	—	—
	> 2	0.964 (0.909–1.024)	0.239	0.915 (0.684–1.225)	0.553
Ever breastfed	No^a^	—	—	—	—
	Yes	0.977 (0.885–1.078)	0.648	0.947 (0.565–1.587)	0.838
Gender of preceding child	Boy^a^	—	—	—	—
	Girl	1.019 (0.960–1.082)	0.521	1.310 (0.988–1.737)	0.160
Considering importance of spacing	No^a^	—	—	—	—
	Yes	1.118 (0.266–4.691)	0.879	1.079 (0.784–1.485)	0.639
Previous delivery by C‐section	No^a^	—	—	—	—
	Yes	0.899 (0.845–0.957)	0.001	5.255 (3.862–0.7.149)	0.022
Age of husband	10–19	—	—	—	—
	20–24	10.77 (0.025–45.674)	0.981	0.046 (0.009–0.226)	0.501
	25–29	0.816 (0.008–75.24)	0.930	0.151 (0.035–0.643)	0.211
	30–34	0.560 (0.002–113.30)	0.831	0.232 (0.055–0.972)	0.146
	35–39	0.233 (0.000–408.76)	0.703	0.449 (0.107–1.875)	0.273
	40–44	0.123 (0.000–540.82)	0.625	0.526 (0.114–2.420)	0.410
	45–49	0.110 (0.000–530.22)	0.606	0.567 (0.109–2.940)	0.500
	≥ 50	9.499 (8.311–10.856)	0.901	0.822 (0.309–3.640)	0.543
Age at first marriage	10–14^a^	—	—	—	—
	15–19	0.966 (0.195–4.788)	0.967	0.765 (0.395–5.788)	0.668
	20–24	0.678 (0.101–4.515)	0.668	0.776 (0.101–3.524)	0.586
	25–29	0.639 (0.101–4.515)	0.680	0.546 (0.121–4.312)	0.567
	30–34	0.548 (0.469–0.537)	0.589	0.647 (0.439–0.607)	0.797
	35–39	0.533 (0.324–0.541)	0.532	0.443 (0.234–0.491)	0.643
	40–44	0.510 (0.433–0.525)	0.543	0.210 (0.133–0.425)	0.654
Current contraceptive method	Traditional	—	—	—	—
	Modern	1.009 (0.390–2.608)	0.984	0.999 (6953–1.436)	0.997
Current fertility intention	No	—	—	—	—
	Yes	1.597 (0.080–31.752)	0.759	0.416 (0.141–1.224)	0.112
Currently used contraceptive method	No	—	—	—	—
	Yes	1484 (1.109–1.986)	0.108	1.096 (0.967–1.243)	0.150
Ever used contraceptive method	No	—	—	—	—
	Yes	1.604 (0.905–2.841)	0.105	1.117 (0.824–1.515)	0.474
Spacing intention	No	—	—	—	—
	Yes	0.602 (0.2296–1.582)	0.304	1.059 (0.8117–1.382)	0.672
Interaction effects of mother education and socioeconomics status					
Primary educated mother × Rich socio economic status		1.39	0.058	1.28	< 0.001
Middle educated mother × Rich socio economic status		1.55	0.013	1.54	< 0.001
Highly educated mother × Rich socio economic status		1.93	0.002	2.10	< 0.001
Interaction effect of mother education and region					
Middle educated mother × Rural residence		0.84	0.274	0.87	0.003
Higher educated mother × Rural residence		0.86	3.35	0.88	0.009
Interaction effect of socioeconomic status and region					
Middle socioeconomic status × Rural residence		0.78	< 0.001	0.834	< 0.001
Rich socioeconomic status × Rural residence		—	—	0.962	0.017

Abbreviations: AIRR = adjusted incidence rate ratio; AOR = adjusted odds ratios; CI = confidence interval.

^a^ Reference category.

Table 3 shows the Poisson regression and logistic regression results of the CEB of the women of Punjab, Pakistan, including incidence and Odd rate ratios (AIRR and AOR), and their confidence intervals. The women having age 20–49 (AIRR, 1.369; 95% CI, 1.019–1.838; AIRR, 1.809; 95% CI, 1.359–2.409, AIRR, 2.253; 95% CI, 1.690–3.003; AIRR, 2.565; 95% CI, 1.918–3.430, AIRR, 2.789; 95% CI, 2.050–3.793, AIRR 3.340; 95% CI, 2.196–5.080) was significantly higher expected continuity of childbearing than for women under age aged 15–19 by keeping all the other variables constant. This pattern is consistent with the natural progression of reproductive life, as older women have had a longer period of exposure to the risk of childbearing. Women with primary (AIRR, 0.79; 95% CI, 0.66–0.95), middle (AIRR, 0.75; 95% CI, 0.63–0.91), and higher education (AIRR, 0.67; 95% CI, 0.55–0.81) had significantly lower fertility rates compared with women with no education. Women with higher levels of education tend to have more clearly defined reproductive goals and are less likely to revise their fertility plans over time, in contrast to their less educated counterparts. Women from middle (AIRR, 0.87; 95% CI, 0.80–0.95) and richest (IRR, 0.85; 95% CI, 0.78–0.92) wealth quintiles showed reduced expected numbers of children compared to the poorest group. One plausible explanation is that women in higher socioeconomic strata tend to have higher levels of education and are often employed in demanding professional roles, which may limit the time and resources available for raising larger families. Women who reported that they wanted no more children had a higher expected number of children (AIRR, 1.41; 95% CI, 1.32–1.50) than who reported that later. Consistent with previous findings, this study revealed that women who expressed a desire for more children, whether within a planned timeframe or at a later stage, tended to have fewer children on average compared to those who were uncertain about the timing of future childbearing. According to the 2018 National Health Statistics (National Institute of population studies (NIPS) Pakistan and ICF International 2019) Reports from the United States, fertility intentions vary notably across racial and religious groups, and such variations are often shaped by broader sociocultural contexts. One of the existing study demonstrated that initial negative plans of child desire showed significant instability of childbearing plans. Women who had a delivery by C‐section showed lower expected fertility than those who delivered normally (AIRR, 0.90; 95% CI, 0.85–0.96).

The result of logistic regression shows that Women aged 25–29 years had higher odds of having more than two children compared with those aged below 20 (AOR, 11.93; 95% CI, 2.49–57.04). The likelihood of higher fertility increased further with age, as women aged 30–34 years (AOR, 20.18; 95% CI, 4.17–97.76) and 35–39 years (AOR, 43.92; 95% CI, 8.49–227.19) were significantly more likely to have more than two children. This pattern is consistent with the natural progression of reproductive life, as older women have had a longer period of exposure to the risk of childbearing. Women with higher education (AOR, 0.18; 95% CI, 0.05–0.70) had lower odds of having more than two children compared to those with no education (AOR, 0.18; 95% CI, 0.05–0.70). Women with higher levels of education tend to have more clearly defined reproductive goals and are less likely to revise their fertility plans over time, in contrast to their less educated counterparts. Similarly, women from richer households were less likely to have more than two children (AOR, 0.495; 95% CI, 0.31–0.78). One plausible explanation is that women in higher socioeconomic strata tend to have higher levels of education and are often employed in demanding professional roles, which may limit the time and resources available for raising larger families. Women who delivered their last child by casarean section were less likely to have more than two children compared with those who delivered normally (AOR, 0.72; 95% CI, 0.54–0.95). Women who reported not wanting more children had higher odds of having more than two children than those who still wanted additional children (AOR, 5.26; 95% CI, 3.86–7.15). Consistent with previous findings, this study revealed that women who expressed a desire for more children, whether within a planned timeframe or at a later stage, tended to have fewer children on average compared to those who were uncertain about the timing of future childbearing. According to the 2018 National Health Statistics Reports from the United States, (National Institute of population studies (NIPS) Pakistan and ICF International 2019) fertility intentions vary notably across racial and religious groups, and such variations are often shaped by broader sociocultural contexts. One of the existing study demonstrated that initial negative plans of child desire showed significant instability of childbearing plans.

Significant interaction effects indicate that the protective effect of education is modified by socioeconomic status. Specifically, middle‐educated women in the richest households had higher odds of high fertility (OR = 1.55; 95% CI = 1.09–2.18; *p* = 0.013), and higher‐educated women in the richest households had the strongest increase in odds (OR = 1.93; 95% CI = 1.27–2.91; *p* = 0.002), suggesting that wealth can weaken or reverse the fertility‐reducing effect of education. Also in poisson regression model significant interactions indicate that the fertility‐reducing effect of education is weakened among wealthier households. Specifically, primary‐educated women in the richest households had IRR = 1.28 (95% CI = 1.16–1.42; *p* < 0.001), middle‐educated women in the richest households had IRR = 1.54 (95% CI = 1.39–1.72; *p* < 0.001), and higher‐educated women in the richest households had IRR = 2.10 (95% CI = 1.82–2.42; *p* < 0.001). Non‐significant interactions (e.g., primary or middle education with middle wealth, higher education with middle wealth) suggest that education's protective effect is stable in lower‐wealth households.

Significant interactions for middle and higher education with rural residence indicate that the fertility‐reducing effect of education is slightly stronger in rural areas, while primary education has a similar effect in both urban and rural settings.

However, the interaction between middle socioeconomic status and rural residence is significant (OR = 0.78; *p* < 0.001), indicating that middle socioeconomic status women in rural areas experience a stronger reduction in high fertility than expected from main effects alone. Significant interactions indicated that the fertility‐reducing effect of wealth is slightly stronger in rural settings.

Table 4 indicates a comparison of adequacy of the fitted models in child ever born as binary logistic and poisson count model. Hosmer–Lemeshow test, Pearson‐*χ* ^2^, and Deviance goodness‐of‐fit showed that fitted models for child ever born are insignificant at *p* > 0.05. This indicates that both models are best fitted.

**TABLE 4:** Summary Statistics of diagnostic tests of both models.

Tests vs. logistic regression models	Child ever born logistic model $\chi^{2}$(*p*)	Tests vs. Poisson models	Child ever born Poisson model $\chi^{2}$(*p*)
Pearson–*χ* ^2^ test	653.35 (0.4013)	Pearson–*χ* ^2^ test	589.4955 (1.0000)
Hosmer–Lemeshow test	2.32 (0.9697)	Deviance goodness‐of‐fit	586.7643 (1.0000)

**FIGURE 1:**
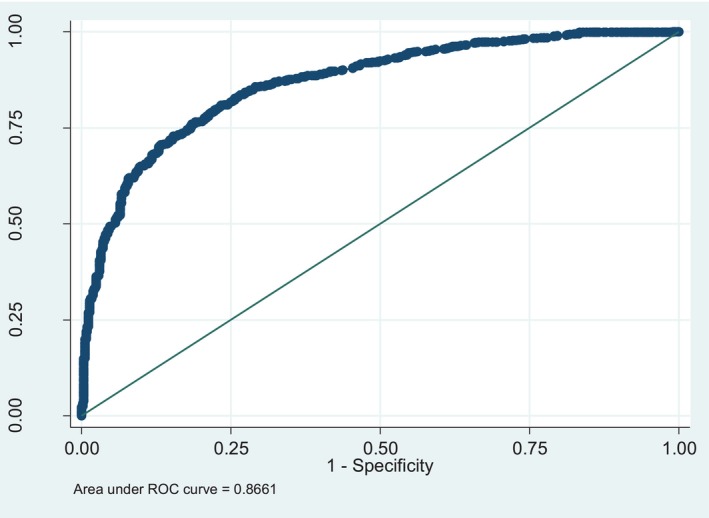
Receiver operating curve of binary logistic model.

For both separate models, sensitivity and specificity classification and receiver operating curves can be used to assess the quality of prediction of fitted models. Area under the receiver operating curve is used to check the total discriminating power of the binary logistic regression model. This is the graph between 1‐specificity on the *x*‐axis and sensitivity on the *y*‐axis. The ROC curve, closer to the upper left diagonal, gives better performance. Here, from Figure 1 it has been shown that the curve lies in the upper left corner; therefore, the total area under the receiver operating curve is 86.61% for child ever born (binary logistic model).

Table 5 provided the useful information about classification of fitted binary logistic model. Overall correct classification of Child ever born is 79.61%.

**TABLE 5:** Sensitivity and specificity classification of child ever born.

Observed	Predict		
	Yes (CEB = 1)	No (CEB = 0)	Percent correct
Yes (CEB = 1)	736	119	86.11%
No (CEB = 0)	163	363	69.01%
Overall	79.61%		

## 4 Discussion

The number of CEB is commonly used as a measure of lifetime fertility. Fertility remains a fundamental demographic factor, critical for the continuity of human populations. For policymakers, understanding fertility patterns is essential to inform the development and implementation of effective family planning programs. In the context of Pakistan, rapid population growth poses a significant challenge to the country's economic development. Although various population control policies have been introduced over the years, Pakistan has only recently entered the early transitional phase of fertility decline. To ensure timely and effective population policy planning, it is vital to examine the underlying dynamics of fertility behavior. This study specifically explored the stopping behavior of fertility reflected in the total number of CEB within a sociodemographic framework. The results indicated that advancing maternal age is significantly associated with higher expected childbearing. In contrast, factors such as a later on desire of more children, previous delivery by C‐section, and rich wealth status of woman and higher education of woman were all significantly associated with lower expected fertility levels and childbearing.

Maternal age is widely recognized as one of the most influential biological and demographic determinants of fertility. It plays a pivotal role in shaping the number of CEB to a woman. The findings of this study indicate that women aged 45–49 had significantly more children compared to those in younger age groups, beginning from age 15. This pattern is consistent with the natural progression of reproductive life, as older women have had a longer period of exposure to the risk of childbearing. A positive association between maternal age and CEB was observed, which aligns with findings reported in previous studies (Bwalya and Odimegwu 2025; Kiser and Hossain 2019; Gebre 2024; Saleem et al. 2025).

In contrast, a significant inverse relationship was found between household economic status and children ever born. Women from wealthier households had fewer children than those from lower‐income backgrounds. One plausible explanation is that women in higher socioeconomic strata tend to have higher levels of education and are often employed in demanding professional roles, which may limit the time and resources available for raising larger families. Studies posit that wealthier parents opt to invest more in fewer children, prioritizing their education and well‐being. However, additional sociocultural factors may also influence this relationship in developing countries such as Pakistan. For instance, wealthier families may prefer to have fewer children to ensure equitable distribution of property and preserve family wealth. In contrast, families in lower socioeconomic groups, particularly those residing in rural areas and dependent on agriculture, often value larger families. In such settings, children are viewed as economic assets who can contribute to household income through labor. The prevalence of child labor and reliance on family labor in agriculture reinforce this preference. These findings are consistent with previous research conducted in similar socioeconomic co3texts (Kiser and Hossain 2019; Gebre 2024; Karimuzzaman et al. 2020; Al‐Balushi et al. 2020).

Desire for more children was found to be significantly associated with the number of CEB. Consistent with previous findings, this study revealed that women who expressed a desire for more children, whether within a planned timeframe or at a later stage, tended to have fewer children on average compared to those who were uncertain about the timing of future childbearing. According to the 2018 National Health Statistics Reports from the United States, fertility intentions vary notably across racial and religious groups, and such variations are often shaped by broader sociocultural contexts. One of the existing studies demonstrated that initial negative plans of child desire showed significant instability of childbearing plans (Bonanni et al. 2025; Ibupoto, Shah, Loong 2025).

Education plays a critical mediating role in shaping fertility intentions and CEB. Women with higher levels of education tend to have more clearly defined reproductive goals and are less likely to revise their fertility plans over time, in contrast to their less educated counterparts. Numerous studies have highlighted the significant influence of women's education on both fertility intentions and actual reproductive outcomes, including the total number of CEB. These findings underscore the importance of promoting female education as a strategy not only for improving reproductive autonomy but also for managing fertility levels at the population level (Bwalya and Odimegwu 2025; Gebre 2024; Afreen et al. 2024; Saleem et al. 2025; Shah et al. 2025; Ibupoto, Shah, Sang 2025).

These findings align with demographic transition theory, which suggests that fertility declines with socioeconomic advancement and modernization. In the context of Punjab, Pakistan, the lower fertility observed among educated women and those from wealthier households reflects the ongoing transition toward smaller family sizes and a growing emphasis on child wellbeing and education. From a feminist perspective, increased access to education and greater decision‐making autonomy among women in Punjab contribute to delayed marriage and enhanced control over reproductive choices, thereby reducing fertility levels. Furthermore, the socioeconomic factors of fertility indicate that improved living standards, healthcare access, and awareness evident in the association between casarean deliveries and lower fertility shape reproductive behavior. Collectively, these frameworks underscore how socioeconomic and cultural dynamics specific to Punjab influence fertility outcomes within the broader demographic transition in Pakistan.

To effectively address fertility differentials in Punjab, Pakistan, policy measures should be grounded in demographic transition, feminist, and socioeconomic perspectives.

First, government and development partners should prioritize expanding girls' access to quality education and strictly enforce laws prohibiting child and early marriage. Such initiatives would help delay the onset of childbearing and promote informed reproductive decisions. Second, policies aimed at enhancing women's economic participation through vocational training, microfinance schemes, and employment opportunities can strengthen women's autonomy and reduce fertility levels. Third, scaling up family planning and reproductive health services, particularly in rural and low‐income areas, is essential to reduce unmet contraceptive needs.

Fourth, leveraging digital innovations, including mobile health platforms and social media campaigns, can enhance awareness and accessibility of reproductive health information.

Finally, integrating gender empowerment objectives into national population and development frameworks is vital to address structural inequalities and sustain fertility decline.

Collectively, these measures call for a multi‐sectoral policy approach that links education, gender equality, and economic empowerment to population management and sustainable development in Pakistan.

## 5 Study Limitations

This study is subject to several data‐related limitations. The dataset lacked detailed information on individual fertility preferences, patterns of social interaction, and key biological determinants such as postpartum amenorrhea, sexual abstinence, and other proximate fertility indicators. Additionally, variables related to media exposure, contraceptive use frequency, and duration of contraceptive use were not available in the dataset, limiting a more nuanced understanding of their impact on fertility outcome child ever born. Despite these limitations, the study offers robust empirical insights grounded in rigorous methodology and a nationally representative sample, contributing valuable evidence to inform policies aimed at managing fertility levels, particularly in relation to first births.

## 6 Conclusions

The study identified several maternal and sociodemographic characteristics associated with the number of CEB. Advanced maternal age was positively correlated with higher fertility, whereas higher educational attainment, delayed fertility intentions, rich economic status of household, and prior casarean delivery were all negatively associated with total number of child ever born. These findings highlight critical areas for policy intervention, particularly, in designing and refining strategies to reduce large family sizes. Strengthening family planning services and enhancing awareness through educational and economic empowerment initiatives may be effective in controlling child ever born rates and curbing population growth. The inverse relationship between maternal education and the number of CEB underscores the need for continued investment in female education. Policies that aim to increase educational attainment, particularly, secondary and higher education for girls can play a pivotal role in reducing fertility rates by enhancing women's decision‐making autonomy and awareness of reproductive health options. Policy interventions should prioritize equitable access to contraception and reproductive health education for women in low‐income and rural communities, where fertility tends to be higher. In conclusion, an integrated approach that combines education, economic empowerment, equitable access to reproductive health services, and age‐sensitive strategies is essential for managing fertility levels and supporting women's reproductive choices. These policy directions will not only help in controlling population growth but also contribute to broader social and economic development goals.

## Author Contributions

**Maryam Siddiqa:** conceptualization, methodology, data curation, formal analysis, writing original draft. **Amber Zubair:** literature review, data collection, visualization, formal analysis, writing review and editing. **Asif Hanif:** statistical analysis, software, validation, data interpretation, writing initial and final draft. **Shazia Iqbal:** methodology, validation, writing initial and final draft. **Tahira Ashraf:** investigation, data verification, writing, review and editing final draft. **Shahzad Ahmad:** critical revision, methodology, writing initial and final draft. All authors reviewed the final draft and gave final approval of manuscript.

## Funding

The authors have nothing to report.

## Disclosure

The study involved secondary data analysis, and no direct involvement with human participants occurred. Therefore, patient consent was not required. Authorization to access and use the Pakistan Multiple Indicator Cluster (PMICS) datasets was granted upon a formal request submitted through their online data access.

## Ethics Statement

This study was conducted in accordance with the ethical principles outlined in the Declaration of Helsinki. Ethical clearance was obtained from the Institutional Review Committee of the Islamic International University, Islamabad, Pakistan (IIU ORIC Bioethics Committee; Protocol No. 110, dated April 22, 2022).

## Consent

The authors have nothing to report.

## Conflicts of Interest

The authors declare no conflicts of interest.

## Data Availability

The dataset analyzed during this study is publicly accessible online on the following link: http://mics.unicef.org/surveys.
